# Hospitalizations and Deaths Associated with *Clostridium difficile* Infection, Finland, 1996–2004^1^

**DOI:** 10.3201/eid1505.081154

**Published:** 2009-05

**Authors:** Outi Lyytikäinen, Heli Turunen, Reijo Sund, Marja Rasinperä, Eija Könönen, Petri Ruutu, Ilmo Keskimäki

**Affiliations:** National Public Health Institute, Helsinki, Finland (O. Lyytikäinen, M. Rasinperä, P. Ruutu, E. Könönen); National Research and Development Centre for Welfare and Health, Helsinki (H. Turunen, R. Sund, I. Keskimäki)

**Keywords:** Enteric infections, bacteria, Clostridium difficile, epidemiology, incidence, mortality, case fatality, population-based, register-based, research

## Abstract

To determine whether the rate of *Clostridium difficile*–associated disease (CDAD) and CDAD-related deaths were increasing in Finland, we analyzed registry data from 1996 through 2004. We determined the number of hospital discharges that had a diagnosis code specific for CDAD from the International Classification of Diseases, 10th revision: “enterocolitis due to *Clostridium difficile”* (A04.7) and “pseudomembranous enterocolitis associated with antimicrobial therapy” (K52.8), listed as any diagnosis in the National Hospital Discharge Registry. CDAD-related deaths were identified from death certificates. Those discharged with a CDAD diagnosis doubled from 810 (16/100,000 population) in 1996 to 1,787 (34/100,000) in 2004. The increase was most prominent for patients >64 years of age but concerned only those discharged with diagnosis code A04.7. The number of those discharged with diagnosis code K52.8 remained stable. The age-standardized mortality rate associated with CDAD increased from 9/million in 1998 to 17/million in 2004; the increase was limited to persons >64 years of age.

## Abstract 2

The age-standardized mortality rate associated with CDAD increased from 9 per million in 1996 to 17 per million in 2004 among persons >64 years of age.

Toxin-producing *Clostridium difficile* is the most frequent cause of antimicrobial drug–associated diarrhea. The clinical spectrum of *C. difficile*–associated disease (CDAD) ranges from mild diarrhea to severe life-threatening pseudomembraneous enterocolitis. Several reports from hospitals in the United States, Canada, and Europe suggest that the incidence and severity of CDAD are increasing (*1*). This increase is assumed to be associated with higher toxin production by a fluoroquinolone-resistant strain belonging to pulsed-field gel electrophoresis type NAP1, PCR ribotype 027, and toxinotype III (*2*,*3*). However, the same virulence factors may also be present in other *C. difficile* strains. Since 2003, PCR ribotype 027 has been detected in several European countries, including Finland in 2007 (*4*,*5*). Little information from nationwide, population-based studies on CDAD epidemiology existed before the emergence of this strain.

Various methods and strategies are used to diagnose *C. difficile* infection, which makes it difficult to accurately compare the epidemiology of CDAD over time and place (*6*). Toxin detection, along with culture, is considered the most accurate method of CDAD diagnostics, and it also allows the isolates to be typed in epidemiologic studies.

In Finland, CDAD has not continuously been a notifiable disease. To determine whether the rate of CDAD and CDAD-related deaths were increasing in Finland, we analyzed data from 2 national registers, the hospital discharge and death registers, from 1996 through 2004. We also conducted a national survey to evaluate the methods used in clinical microbiology laboratories in Finland to diagnose infection with *C. difficile*.

## Methods

In Finland (population 5.3 million), the national healthcare system is organized into 20 geographically and administratively defined healthcare districts; populations range from 67,800 to 1.7 million. Fifteen healthcare districts have only secondary and primary care hospitals, and 5 also provide tertiary care services.

### Data Sources

The National Hospital Discharge Registry (HILMO) contains comprehensive healthcare records on inpatients, provided by all hospitals and municipal health centers in Finland. Each report to HILMO includes the patient’s national identity code, admission and discharge dates, healthcare provider, type of service, medical specialty, the place (home or institution) from which the patient was transferred to the facility, and data on surgical procedures and discharge diagnoses. HILMO data were used to determine the number of discharges with a code specific for CDAD from the International Classification of Diseases, revision 10 (ICD-10): “enterocolitis due to *Clostridium difficile”* (A04.7) and “pseudomembranous enterocolitis associated with antimicrobial therapy” (K52.8), listed as the first or any discharge diagnosis. CDAD-related deaths were identified from death certificates of the National Population Information System in which underlying and immediate causes of death and contributory factors are currently coded according to ICD-10. In addition, for patients with CDAD identified from HILMO, we obtained death certificate data by using the national identity code, and, correspondingly, for persons with CDAD identified from death certificates, we obtained their discharge register data in HILMO for the preceding year.

### Incidence, Mortality Rates, Case-Fatality Rates, and Statistical Analysis

Data from the National Population Information System for years 1996–2004 were used as denominators to calculate age- and sex-specific incidence and mortality rates. The average annualized incidence rates during the surveillance period in different healthcare districts were calculated by using the total number of discharges within the total population during 1996–2004. For age standardization, we used the European Standard Population as appropriate (*7*). To evaluate secular trends, we calculated the rates for different groups, classified by age and sex, for each 12-month period from January 1996 through December 2004. Poisson regression was used to assess whether the observed changes in the rates were statistically significant. We calculated case-fatality proportions by dividing all deaths due to any cause within 30 days after the last CDAD-related discharge, including CDAD-related deaths, by the total number of patients discharged with a CDAD-related diagnosis. Appropriate permissions to use the data from the national hospital discharge and death registers were acquired from Statistics Finland and the National Research and Development Center for Welfare and Health.

### Laboratory Survey

In June 2006, we sent a standardized questionnaire to 32 clinical microbiology laboratories, which represented all tertiary (n = 5) and secondary (n = 16) care hospital laboratories and most private laboratories in Finland. The questionnaire covered different aspects pertaining to CDAD diagnosis, such as requests and criteria used for undertaking *C. difficile* investigations, current methods used for the diagnosis, and changes in diagnostic procedures during 1996–2005 as well as the total number of investigations that identified *C. difficile* in 2005.

## Results

We found 7,946 and 10,958 discharges from 1996 through 2004, for which CDAD was listed as either the primary diagnosis or as any diagnosis, respectively. Among 8,093 individual patients, 5,239 (65%) were >64 years of age and 5,005 (62%) were female. The average annualized age-specific incidence rate was >6-fold higher for patients >64 years of age than for those 45–64 years of age (108 vs. 16 hospital discharges/100,000 population). The age-standardized rate was similar for female and male patients (19/100,000 population versus 18/100,000).

**Figure 1:**
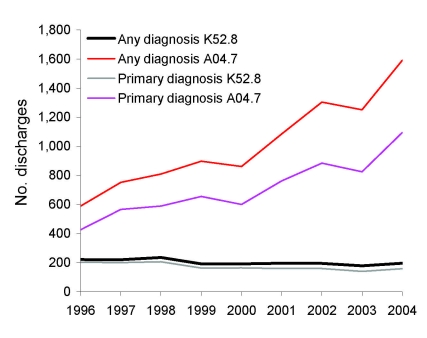
Number of hospital discharges with *Clostridium difficile* listed as any and primary diagnoses, 1996–2004, Finland. International Classification of Diseases, 10th revision, codes K52.8, “pseudomembranous enterocolitis associated with antimicrobial drug therapy,” and A04.7, “enterocolitis due to *Clostridium difficile*.”

**Figure 2:**
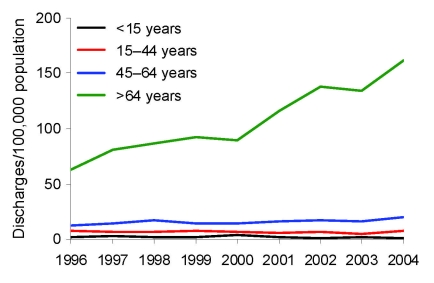
Rates of hospital discharges with *Clostridium difficile* listed as any diagnosis, by age, 1996–2004, Finland.

Discharges for which CDAD was listed as any diagnosis doubled from 810 (16/100,000 population) in 1996 to 1,787 (34/100,000 population) in 2004 (Figure 1). The number of instances in which a patient was given a first diagnosis of CDAD also increased. The increases only concerned discharges with code A04.7; the number of discharges with code K52.8 remained at the same level. The increase was most prominent for patients >64 years of age, from 63/100,000 population to 162/100,000 (Figure 2). A slight increase was detected for those 45–64 years of age but none for children or those 15–44 years of age. The numbers of CDAD-related discharges increased in most healthcare districts (80%, 16/20) and in all tertiary care regions (5/5); the trend was statistically significant in 13 of the 20 (65%) healthcare districts (p<0.05 by Poisson regression). The average annualized age-standardized incidence rates varied greatly between the healthcare districts, from 9/100,000 population to 28/100,000.

**Figure 3:**
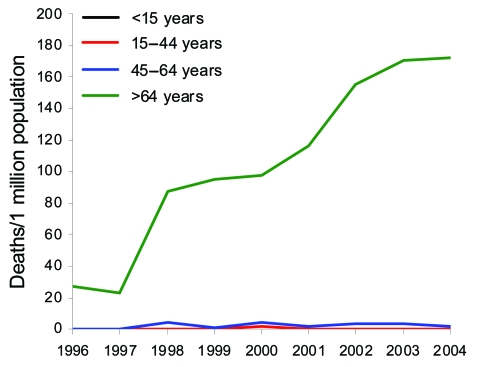
Mortality rates associated with *Clostridium difficile,* by age, 1996–2004, Finland.

A total of 761 CDAD-related deaths were identified (range by year, 18–143); most (733/761, 96%) occurred in persons >64 years. For 81 patients (11%) CDAD was considered as the underlying cause of death (range by year, 7–13). During 1996–1997, the annual number of CDAD-related deaths was ≈20, and thereafter the number increased from 70 to a peak of 140. During 1998–2004, the age-standardized mortality rate associated with CDAD increased from 9 per million population to 17 per million. The increase in mortality rate was limited to persons >64 years of age; during 1998–2004, the age-specific mortality rate of CDAD for persons >64 years of age doubled, from 76 per million population to 146 per million (Figure 3).

Of the 8,402 patients discharged with a CDAD-related diagnosis, 1,196 (14.2%) died within 30 days. The overall case-fatality proportion was similar for female and male patients (14.1% vs. 14.4%) but varied by age group; the case-fatality proportion was highest for patients >64 years (20.5%) and lowest for those 15–44 years (0.8%). From 1996 through 2004, the case-fatality proportion doubled, from 7.6% to 15.5%.

Of the 32 clinical microbiology laboratories invited to take the survey on diagnostics, 28 laboratories (88%) responded, including all healthcare districts except 1 (19/20, 95%). Twenty-four (86%) laboratories, in 18 different healthcare districts, reported routinely performing *C. difficile* diagnostic tests. All laboratories had specific requests for *C. difficile* investigation or for investigating cases of antimicrobial drug–associated diarrhea. The methods used included toxin detection (88%) and culture (79%); most laboratories currently detect both toxins A and B by commercial kits, and 3 detect only toxin A. In 16 laboratories, a positive result reported to clinicians was based on both a positive culture and a toxin test result; in 5, on a positive toxin test result, and in 3, on a positive culture alone. In most laboratories, the change from toxin A detection only to testing for both toxins A and B occurred after the year 2000. In 2005, diagnostic sampling activity varied nearly 10-fold between healthcare districts, from 209 to 1,845 tests per 100,000 population. The laboratories also had wide variation in the proportion of samples positive for *C. difficile* (1%–28%), as well as in those positive for toxin (range 3%–15%).

## Discussion

Our nationwide population-based study shows that the rates of CDAD and CDAD-related deaths among elderly patients are increasing in Finland. However, on the basis of ICD-10 codes, we did not detect an increase in the severe form of CDAD, pseudomembranous enterocolitis. Although the rates of CDAD and CDAD-related deaths in Finland are still lower than the population-based estimates reported from the United States (*8*) and the United Kingdom (*9*), clinicians in Finland were reminded about the increasing risk for CDAD, especially among elderly patients.

A similar population-based study has been performed in the United States, where the number of hospital patients discharged with CDAD listed as any diagnosis doubled from 1996 through 2003 (*8*). The most pronounced increase occurred during 2000–2003. The US incidence increased to a considerably higher level than that in Finland (61 discharges/100,000 population vs. 34/100,000); the US increase was several-fold among elderly patients, as in Finland. A difference from our study was that ICD-9 codes were in use in the United States during the study period, and only code 008.45 (intestinal infection caused by *C*. *difficile*) was analyzed. A doubled rate was also found in another US study, which was limited to adults hospitalized with CDAD during 2000–2005 (*10*).

The CDAD-related mortality rate has been evaluated in England and Wales using death register data (*9*). Deaths associated with CDAD or pseudomembranous enterocolitis more than doubled from an age-standardized mortality rate of 11 per million population in 1999 to 24 per million in 2004. The increase was slightly greater than in Finland during the same period (from 9 to 17 per million population ), and the mortality rate increased to a somewhat higher level; on more than half of the death certificates in England and Wales, CDAD was the underlying cause of death. By 2006, the mortality rate had further increased by 72% to 64–65 per million population (*11*). A similar trend in CDAD-related mortality rates has also reported from the United States during 1999–2004 (*12*). However, those figures are not comparable to ours because the 2000 US population was used as a standard.

In our study, the 30-day case-fatality proportion (deaths from all causes) was 14%, which is similar to findings from other studies that report case-fatality rates ranging from 11% to 25% during hospitalization (*3*,*13*–*16*). However, the results are not directly comparable because we analyzed the deaths within 30 days after the date of discharge with a CDAD-related diagnosis, not after the date of diagnosis, because the latter was not available.

During 1996–2005, considerable changes occurred in Finland in the diagnostic methods of *C. difficile,* which may have contributed to the increasing rate of CDAD. The regional variation in diagnostic activity may also have had an effect. Improvement in CDAD diagnostic procedures can still be made, if culture and toxin detection together are considered the most accurate method.

Our study, as other register-based studies, has some limitations. First, we only analyzed hospital discharge data, and the sensitivity and specificity of hospital discharge coding for CDAD are not well known. In the US study, the correlation between toxin assay result and ICD-9 code was good: the specificity of toxin assay results was excellent (99.7%), but the sensitivity was lower (78%) (*17*). Alternatively, it is very unlikely that the sensitivity would have remarkably changed during the study period, as stated in article about the US study. The validity of Finnish hospital discharge and death registers has been assessed only for diseases other than CDAD (*18*,*19*). The second code we used (K52.8, pseudomembranous enterocolitis associated with antimicrobial therapy) is not specific for *C. difficile,* and its use might have been inconsistent for the severe form of CDAD, which is likely to obscure a possibly increasing trend. Second, because the ICD codes were given at the time of hospital discharge, we could not assess which proportion of CDAD was related to ambulatory healthcare or to in-hospital care. In addition, the changes in laboratory diagnostics may have influenced the results. A considerable proportion of the microbiology laboratories changed from using immunoassays that detect only toxin A to using assays that detect both toxins A and B. That this would be the only reason for the increase in CDAD is unlikely because only a minor proportion of CDAD (5%) has been reported to be caused by strains producing only toxin B (*20*). No such information is available from Finland. In addition, trends in diagnostic activity could not be analyzed because the data were available only for 2005.

According to the first national prevalence survey performed in 2005 in 30 acute care hospitals in Finland, 5% of the healthcare-associated infections confirmed microbiologically were caused by *C. difficile* (*21*). CDAD was >10× more prevalent than infections due to methicillin-resistant *Staphylococcus aureus*. During 1995–1997, findings positive for *C. difficile* (positive culture and/or toxin production) were included in national laboratory-based surveillance reports in Finland. Because electronic reporting was not yet common, surveillance was halted due to the workload and difficulties in interpreting surveillance data because most of the positive findings were based on cultures only.

Our data from this study offer a comprehensive picture of the trends and the outcome of CDAD in a well-defined population during the period before the new virulent PCR ribotype 027 was detected in Finland. This information serves as a point of reference for Finland and other industrialized countries when assessing the effects of emerging, highly virulent clones on the epidemiology of CDAD. Notably, the results we detected in the epidemiology of CDAD are similar to those found in limited settings in many industrialized countries, even though the rates of CDAD and CDAD-related deaths were at a lower level in Finland. As elsewhere, the increase in incidence in Finland may be due to a growing population at risk, reflecting the increased prevalence of chronic disease and improved survival for patients with severe chronic underlying diseases as well as increasing use of antimicrobial drugs. The results of our study have been used to set up a national surveillance system for CDAD, including recommendations for CDAD diagnostic procedures.
